# White matter properties in fronto-parietal tracts predict maladaptive functional activation and deficient response inhibition in ADHD

**DOI:** 10.1038/s41598-025-02326-y

**Published:** 2025-06-06

**Authors:** Daniel Smullen, Andrew P. Bagshaw, Lilach Shalev, Shlomit Tsafrir, Tamar Kolodny, Carmel Mevorach

**Affiliations:** 1 Centre for Human Brain Health and School of Psychology, University of Birmingham, Birmingham, UK; 2 Constantiner School of Education and the Sagol School of Neuroscience, Tel-Aviv University, Tel-Aviv, Israel; 3 Clalit Health Services, Tel-Aviv, Israel; 4 Department of Cognitive Sciences, The Hebrew University of Jerusalem, Jerusalem, Israel; 5 Department of Psychology, University of Washington, Seattle, WA US; 6 Department of Psychology, Ben-Gurion University of the Negev, Be’er Sheva, Israel

**Keywords:** Cognitive neuroscience, Physiology

## Abstract

Response inhibition is a key characteristic of adaptive human behaviour. However, in attention deficit hyperactivity disorder (ADHD) it is often impaired. Previous neuroimaging investigations implicate a myriad of brain networks in response inhibition, making it more difficult to understand and overcome response inhibition difficulties. Recently, it has been suggested that a specific fronto-parietal functional circuitry between the inferior frontal gyrus (IFG) and the intraparietal sulcus (IPS), dictates the recruitment of the IPS during response inhibition in ADHD. To ascertain the critical role of the IFG-IPS functional circuit and its relevance to response inhibition in ADHD, it is crucial to understand the underlying structural architecture of this circuit so that the functional relevance could be interpreted correctly. Here we investigated the white matter pathways connecting the IFG and IPS using seed-based probabilistic tractography on diffusion data in 33 ADHD and 19 neurotypicals, assessing their impact on both IPS recruitment during response inhibition and on response inhibition performance in a Go/No-go task. Our results showed that individual differences in the structural properties of the IPS-IFG circuit, including tract volume and diffusivity, were linked to IPS activation and even predicted response inhibition performance outside the scanner. These findings highlight the structural-functional coupling within the IFG-IPS circuit in response inhibition in ADHD and suggest a structural basis for maladaptive functional top-down control in deficient inhibition in ADHD.

**Supplementary Information:**

The online version contains supplementary material available at 10.1038/s41598-025-02326-y.

## Introduction

 Inhibiting responses is a survival necessity, from holding our tongue to stay out of trouble to not stepping into the road as a car hurtles round the bend. Nevertheless, impaired response inhibition is one of the most prominent and reproducible behavioural dysfunctions in attention deficit hyperactivity disorder (ADHD^1–4^),, and can be linked to various detrimental outcomes in the condition such as increased risk of school suspension^5^, reduced job stability^6^, increased risk of injury and increased risk of substance abuse^7^. However, a full mechanistic understanding of the neurobiological mechanisms underlying response inhibition in ADHD is still lacking.

Neuroimaging studies have pointed to a large-scale network of frontal, parietal and striatal brain areas, which is recruited during response inhibition tasks. Prominent regions are the inferior frontal gyrus (IFG), insular cortex, supplementary and pre-supplementary motor areas, temporal and parietal areas, the basal ganglia, and the subthalamic nucleus^8–13^. Various studies demonstrated reduced activity in these regions in participants with ADHD (for meta-analyses see^14–17^, as well as alterations of the functional connectivity between them^18–20^.

However, the recruitment of many of the aforementioned areas is not specific to inhibition per-se, and can be attributed to other visual, motor and cognitive demands involved in response inhibition tasks^21–27^. Recently, using a Go/No-go task with a manipulation of target frequency, Kolodny and colleagues^28^ narrowed down these broad frontoparietal activations to specific parietal nodes of the network (bilateral intraparietal sulcus (IPS) and left temporoparietal junction (TPJ)), which were directly modulated by the response inhibition demand^28^. Thus, activity in these parietal nodes dynamically changed (‘parietal modulation’) if the demand for response inhibition was high or low, reflecting their role in supporting cognitive control processes such as inhibition. This increased specificity of response inhibition nodes allowed the authors to identify a marked difference in ADHD^29^, where the IPS and TPJ modulation by inhibitory demand was missing. Moreover, individual differences analysis revealed that the parietal nodes recruitment was mediated by ADHD severity^29^, and that it was likely associated with a similar scaling of the functional connectivity between the IPS and the inferior frontal gyrus (IFG). Thus, inhibitory load was driving both the recruitment of the parietal nodes and the connectivity between parietal and frontal nodes, but this modulation dissipated with increased ADHD severity. These findings provide a mechanistic framework for understanding impaired response inhibition in ADHD, whereby communication between frontal and parietal nodes is less effective and results in reduced sensitivity of parietal nodes to inhibitory demand.

However, the underlying cause for the reduced functional connectivity is still obscure. It could represent deficiencies in the functional activation of the specific nodes, a different cognitive strategy, or a genuine structural connectivity deficit. The latter would manifest in changes to the underlying structural substrate of the white matter (WM) pathways that connect the IFG with the IPS and mediate information transfer between them (Huang & Ding, 2016; Mollink et al., 2019; van den Heuvel & Sporns, 2019). Previous research has highlighted that WM properties appear to differ in ADHD, but this research generally focuses on global WM and there are often discrepancies between findings, reporting both low (Castellanos et al., 2002) and high volume (Seidman et al., 2006) in adults, and children, respectively. Part of the difficulty in interpreting previous studies is related to the focus primarily on childhood ADHD rather than adults, and on whole-brain WM tracts rather than functionally specific ones. Few previous investigations focused on specific tracts which may bear some relevance for the IFG-IPS circuit investigated in the current research, but again this research is limited and inconsistent. For instance, both in corpus callosum pathways as well as within the superior longitudinal fasciculus (SLF) there have been findings of both increased and decreased WM properties (FA and MD) in ADHD compared with controls (Castellanos et al., 2002). Even though these tracts somewhat overlap the IFG-IPS circuit, they are distinct neural pathways. To our knowledge, thus far no research has been conducted on the specific IFG-IPS circuit that is of primary interest to the current research.

We therefore propose that in order to better understand the role of fronto-parietal functional connectivity and activation in response inhibition in ADHD we should assess the WM properties of the specific underlying structural pathway. Utilizing a multi-modal fMRI-dMRI-behavioural dataset, we use the previous findings of maladaptive frontoparietal functional connectivity during response inhibition in ADHD to guide a targeted analysis of the diffusion-weighted (dMRI) data. We employed seed-based probabilistic tractography delineating WM tracts connecting bilateral IFG and IPS to extract volume and microstructural properties along those specific tracts. We first tested whether any of the structural metrics were atypical in ADHD compared to controls. We next assessed if individual differences in the WM properties of these tracts could predict the individual differences in parietal modulation by inhibitory demand (measured with fMRI). Finally, we also tested if these structural properties could predict performance in a response inhibition task recorded outside the scanner.

## Methods

### Participants

The sample consisted of 42 adults with ADHD and 24 neurotypical controls who also participated in our previous studies (*Table S2*)^28–30^. Participants were recruited via advertisements at college and university campuses. All ADHD participants had a previous diagnosis by a qualified clinician, and underwent full psychiatric evaluation conducted by author ST – a certified psychiatrist. The interview included psychiatric history and mental status examination according to DSM-5 criteria. Participants were excluded if they had any neurological or psychiatric disorders other than ADHD, including major depression, anxiety, OCD or psychosis; if they did not currently meet diagnostic criteria for ADHD; or if they were using any non-psychostimulant psychotropic medication. ADHD current symptom severity was estimated using the total score on the Hebrew version of the Adult ADHD Self-Report Scale (ASRS), a short scale comprised of 18 items corresponding to the DSM diagnostic criteria^31^.

Neurotypical participants had no prior history of neurological or psychiatric disorders and no learning disabilities, and were not taking any psychotropic medications, including psychostimulants. To ensure the absence of attention difficulties, they completed the ASRS, and were included only if they scored within 1 SD of the population’s mean^32^.

 Fourteen participants (ADHD = 9; Control = 5) were excluded following dMRI preprocessing due to poor data quality (see Sect. 2.3.2.3). The final sample consisted of 33 ADHD participants and 19 neurotypical participants (Table 1). There was no association between group and sex (x^2^(1,*N* = 52) = 1.942, *p* =.163). Of the ADHD participants, 1 was medication-naive; 5 had used psychostimulant medication in the past; 19 used medication occasionally; and 8 used medication regularly. All participants were required to refrain from using stimulants 24 h prior to the experiment. There was no significant difference in WM metrics between the different medication groups (*Table *S1). The study conformed to the Declaration of Helsinki. This study was approved by the ethics committees of Sheeba Medical Center and of Tel-Aviv University (Israel). All participants provided written informed consent after receiving a complete description of the study.

**Table 1:** Descriptive statistics for demographic and behavioural data.

	ADHD	Control	t	df	*P*	Cohen’s d
Sex (M/F)	17/16	6/13				
Age (years)			0.171	50	0.145	− 0.049
N	33	19				
Range (y)	19–39	23–37				
Mean (y)	27.5	27.7				
SD (y)	4.4	3.3				
ASRS Total (Max 90)			−7.638	50	< 0.001**	2.200
N	33	19				
Range	41–85	23–49				
Mean	61.5	32.2				
SD	12.2	6.9				
Response Inhibition Performance^+^			−1.980	43	0.002*	0.760
N	30	15				
Range	0–0.198	0–0.073				
Mean	0.067	0.033				
SD	0.055	0.016				

^+^Response Inhibition Performance was quantified as the percentage of commission errors during the rare-No-go condition in the behavioural version of the Go/No-go task.

Note: * *p* <.05, ** *p* <.001

Note: SD = standard deviation, df = degrees of freedom

Note: There is variation in the number of participants for each variable due to missing data.

### Response inhibition - Go/No-go task

**Fig. 1:**
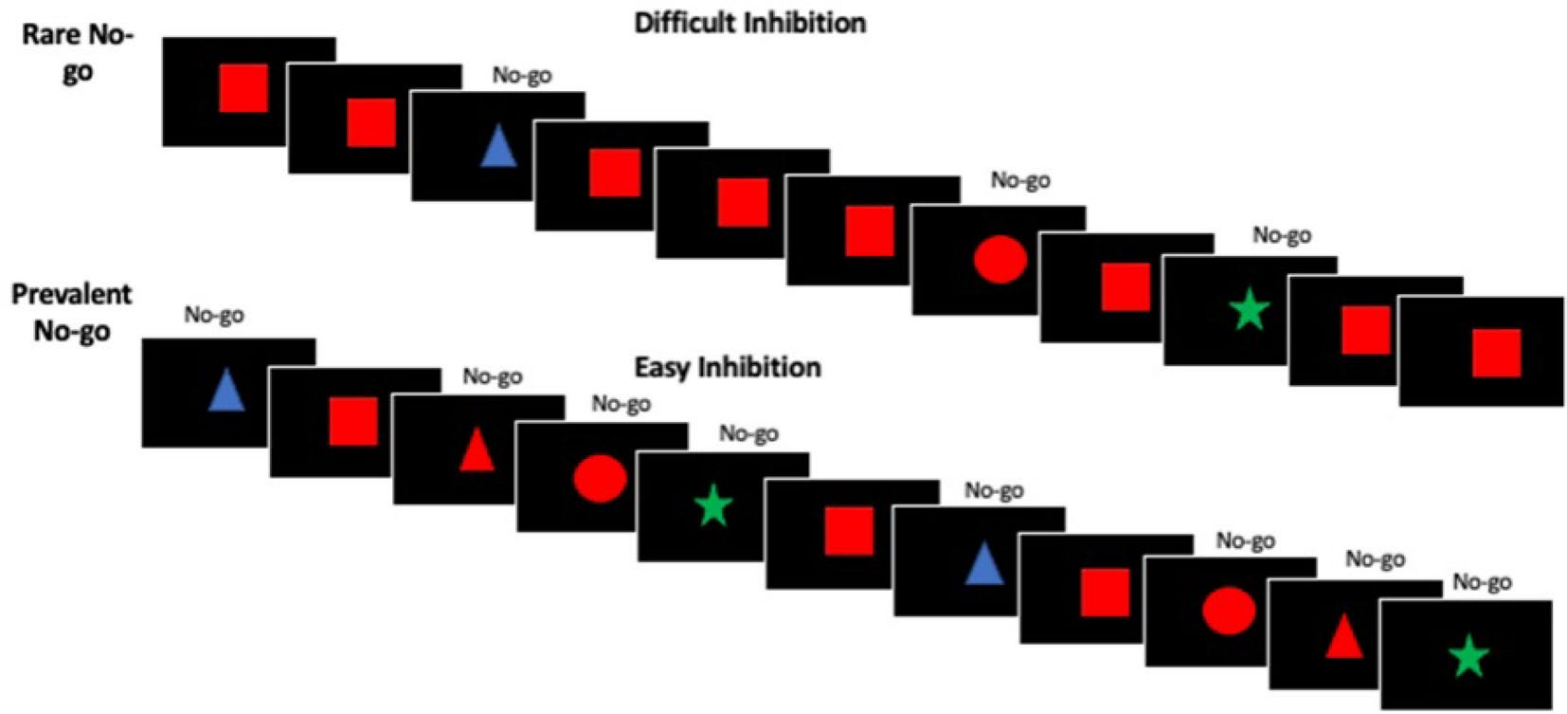
The experimental task, used in the behavioral and fMRI sessions. A series of stimuli were displayed in the centre of the screen. Participants were instructed to respond to a Go stimulus (a red square) using a button click and to ignore all other stimuli (No-go stimuli). There were two task conditions presented in separate runs: rare No-go and prevalent No-go run.

Response inhibition performance was assessed using a Go/No-go task with a manipulation of target frequency (Fig. 1)^28^. Briefly, coloured shapes appeared sequentially in the centre of the screen. Participants were instructed to quickly click a button when a red square appeared (Go trial), and withhold response to any other stimuli (No-go trials). The task consisted of two conditions: rare-No-go and prevalent-No-go. The frequent responses in the rare-No-go condition increase the inhibitory demand for No-go trials relative to the prevalent-No-go condition, and thus by contrasting these conditions the task provides a more clean measure of the cognitive aspect of response inhibition than the historically used version of the task^28^. In the rare-No-go condition, 75% of trials were Go trials whilst 25% were No-go trials. In the prevalent-No-go condition the ratio is inverted so that 25% of trials are Go trials and 75% are No-go trials. In both conditions 1/3 of the No-go trials were same-colour different-shape items, 1/3 were same-shape different-colour items, and 1/3 were different-shape different-colour items. Each stimulus was presented centrally on its own for 100 msec, and the inter-stimulus-interval (ISI) varied from 1.8 s to 12 s, with a mean ISI of 2.75 s. Stimuli and ISI’s were randomly intermixed throughout the block, with a constraint of no more than 3 rare events consecutively. Each block consisted of 164 trials, and lasted a total of 8 min.

The Go/No-go task was used both outside the scanner in a separate behavioural session, and during the fMRI session. The task was identical in both sessions. In the scanner, participants performed 4 runs of the task, two runs of rare-No-go and two runs of prevalent-No-go, interspersed by an anatomical T1-weighted scan. The order of block types was counterbalanced across participants. Response inhibition performance was assessed during the earlier behavioural session due to possible effects of the scanner environment itself on cognitive processes^30,33,34^ and to get an index of performance that reflects trait-like response inhibition. Performance was quantified as the percentage of commission errors made in the rare-No-go condition (i.e., the percentage of failed No-go trials, or false alarms Table 1). Seven participants (ADHD = 3, Controls = 4) were missing behavioural data due to technical failure or time constraints during data acquisition.

## Neuroimaging

Imaging data were collected in a Siemens 3 T Prisma MRI scanner at SCAN@TAU centre in Tel-Aviv University, using a 64-channel head coil. All data were collected within a single scanning session.

### fMRI - parietal modulation by inhibitory demand

fMRI data used in this study were previously published^29^. Detailed methods including acquisition, task, and processing steps are briefly described as they were previously reported. While participants completed the Go/No-go task, functional images were collected using a gradient-echo sequence (see Sect. 2.3.2.3). An ROI analysis was performed to extract parietal modulation by inhibitory demand in right and left IPS and in left TPJ, using masks of inhibition-related activation in neurotypicals^28^. In the ADHD group, percent signal change for each condition (rare-No-go and prevalent-No-go) was computed in reference to an implicit baseline and averaged across voxels within each ROI. For each participant and ROI, the difference between conditions served as the fMRI parietal modulation score (rare No-go minus prevalent No-go), and was used as the outcome measure in the regression models described below. Since the neurotypical group-level data was used to define the ROIs for calculating fMRI modulation scores, extracting individual-level % signal change from these ROIs within the same neurotypical participants would constitute “double-dipping.” This approach would produce non-independent scores, rendering them unsuitable for further analysis. Therefore, fMRI data from the control group was not analysed.

### dMRI

#### dMRI acquisition

dMRI data were acquired using a standard 64-direction protocol using a whole brain echo planar imaging sequence over one phase-encoding direction: phase-encoding direction = A-P, TR = 6900 ms, TE = 53 ms; 76 slices, slice thickness = 1.7 mm, no gap; FOV = 197 × 197 mm, matrix size = 216 × 216, providing a cubic resolution of 1.7 × 1.7 × 1.7 mm. 64 diffusion-weighted volumes (b = 1000 s/mm^2^) and three reference volumes (b = 0 s/mm^2^) were acquired using a standard diffusion direction matrix.

High-resolution T1-weighted anatomical scans were also collected for each participant to aid co-registration, using a magnetization prepared rapid acquisition gradient echo (MPRAGE) protocol (TR = 1.75 s, TE = 2.61 msec, T1 = 900 msec, FOV = 220 × 220, matrix = 220 × 220, axial plane, slice thickness = 1 mm, 160 slices, for an isotropic voxel resolution of 1 mm^3^).

#### dMRI preprocessing

dMRI preprocessing and processing were performed using the FMRIB Diffusion Toolbox (FDT) in FSL^35–37^, following FSL’s standard dMRI processing pipeline. Unweighted (b0) scans were extracted from the dMRI data using the FSL utility tool, fslroi. Brain extraction was performed on one of these images using BET (fractional intensity = 0.5)^38^. Eddy-current and motion correction were performed using EDDY and outlier slices were replaced with slices generated by the Gaussian prediction^39,40^ as this is suggested to be the most effective method of reducing motion artefacts^41^. The number and orientations of crossing fibres within each voxel were modelled using the FDT tool BEDPOSTx. A ball and stick with single diffusion coefficient model was used^42,43^.

#### Quality assurance

The quality of eddy-current correction was assessed using EDDY QUAD and EDDY SQUAD to flag datasets which potentially contained motion artefacts. Datasets were flagged if their absolute and relative motion metrics were outliers when compared to the control group’s distributions of motion metrics, in order to prevent the potential heightened proneness to in-scanner motion in ADHD masking outliers^44^. Flagged datasets were then visually inspected for identifiable motion artefacts. Datasets with a volume containing an identifiable motion artefact were dropped from analysis, as has been previously recommended^45^. This resulted in 8 datasets being excluded (ADHD = 6, control = 2). Whole datasets were excluded rather than individually affected volumes as volume removal risks interfering with diffusion metric estimation^46^. Furthermore, datasets with more than 20% of volumes containing a replaced slice were considered ‘poor quality’ and also dropped from analysis (ADHD = 3, controls = 3)^47^. This resulted in a final dMRI sample of 33 ADHD and 19 controls (Table 1).

#### Probabilistic tractography

**Fig. 2:**
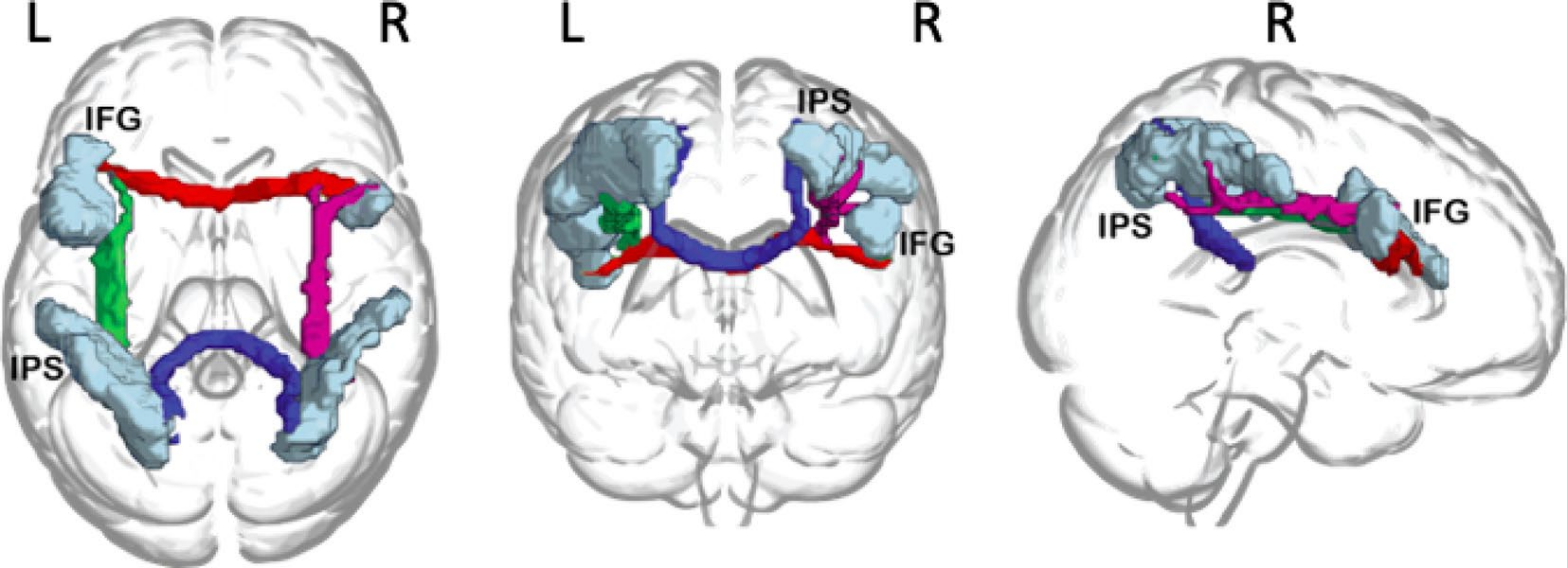
Probabilistic tractography outcomes for an example ADHD participant, warped to standard space and presented in superior, posterior and right views. Seed ROIs in the right and left inferior frontal gyrus (IFG) and left and right intraparietal sulcus (IPS) are shown in light blue. Illustration created by overlaying Probtrackx output on a standard MNI template, using the glass brain setting in MRIcroGL^101^.

ROIs for seed-based tractography were selected at the right IFG, left IFG, right IPS and left IPS, based on the functional connectivity findings in Kolodny et al., (2020). Seed ROIs were defined using the FIND Atlas^48^ and registered to individual subjects’ space using FLIRT^49–51^. Voxel counts for each of the Seed ROIs were as follows: right IFG = 326, left IFG = 1105, left IPS = 2020, right IPS = 1193. Probabilistic tractography was performed using Probtrackx^42,43^ to produce a 3D structural connectivity distribution for white matter tracts connecting the four ROIs. FSL’s standard settings were used: step length = 0.5 mm, samples drawn in each voxel = 5000, subsidiary fibre volume threshold = 0.01, curvature threshold = 0.2. For cross-hemisphere pathways, the corpus callosum was used as a waypoint, defined using the ICBM DTI-81 Atlas^52,53^.The end result of tractography was connectivity distributions for four tracts (Fig. 2): (1) *IFG-IFG*, a tract connecting the right and left IFG; (2) *IPS-IPS*, a tract connecting the right and left IPS; (3) *Right IFG-IPS*, a tract connecting the right IFG and the right IPS; (4) *Left IFG-IPS*, a tract connecting the left IFG and the left IPS.

The IFG-IPS tracts partly overlap with the established Superior Longitudinal Fasciculus (SLF), as determined using the ICBM DTI-81 Atlas. The SLF is a large system of association fibres connecting the frontal and the parietal lobes and prefrontal areas^54–56^. The IFG-IFG or IPS-IPS tracts passed through the genu and splenium of the corpus callosum, respectively, but did not clearly overlap with any major WM pathways from the ICBM DTI-81 Atlas.

#### Diffusion tensor imaging

Diffusion Tensor Fitting was performed at the whole-brain level using DTIFIT to produce Fractional Anisotropy (FA), Axial Diffusivity (AD), Radial Diffusivity (RD) and Mean Diffusivity (MD) values in each voxel. Values from voxels contained within the four tracts described above were averaged to produce tract-level metrics.

#### White matter property extraction

Tract volumes were obtained using fslmaths and fslstats. Spurious tract paths were eliminated prior to volume estimation by thresholding tracts at 15% of maximum path count in each individual and binarizing them to create tract masks^57^. To account for the effects of individual brain size differences on tract volume^58^, for each participant we determined what percentage of that participant’s whole brain volume was accounted for by a tract’s volume. We then determined what volume this percentage accounted for in MNI standard space. The values produced by DTIFIT from voxels contained within the masks of the four tracts described above were averaged to produce tract-level metrics.

To control for outliers, tracts with within-group volume z > 3 were excluded from subsequent analysis for all metrics, on the basis that this likely represents a poor tract reconstruction. FA, AD, RD and MD outliers were excluded individually using the same criterion (i.e., z > 3). Following outlier detection, all IPS-IPS metrics for one control participant were removed from analysis due to the tract’s volume being an outlier. Individually, one control participant’s IFG-IFG MD and AD were also removed from analysis. For the ADHD group, due to tract volume outliers, all metrics for one participant’s IFG-IFG tract were removed from analysis and all metrics for another participant’s IPS-IPS tract were similarly removed.

#### TBSS

As a control measure, we also extracted the global WM metrics using TBSS (Tract-Based Spatial Statistics). The skeleton produced by TBBS is created by first aligning each participants FA image into standard space and then averaging these normalised averages to produce a mean FA map. The mean FA map is thinned so that the skeleton represents the centre of all tracts common to the group.

## Statistical analysis

Parametric tests were carried out in SPSS 26, whilst non-parametric tests were implemented in-house in python. Normality of variables was assessed using the Shapiro-Wilk test of normality^59^, and group differences were tested using t-tests for normally distributed data, and randomisation tests with 100,000 permutations for data with skewed distributions.

To investigate the link between structural connectivity, functional activation and task performance, we used stepwise regression models as this enables the selection of the most important predictors whilst also reducing overfitting by rejecting less relevant predictors. Individual differences are crucial to investigate, as white matter tracts were reconstructed within each participant’s brain, and metrics were calculated at the individual level. By utilising regression models, this approach emphasizes variations between individuals rather than relying solely on group-level comparisons. The five dMRI metrics (volume, FA, RD, MD & AD) of the four tracts (IPS-IPS, right IPS-IFG, IFG-IFG, left IPS-IFG) were used as potential predictors in these models. Parietal fMRI modulation by inhibitory demand, as well as behavioural response inhibition performance, served as outcome measures. For parietal fMRI modulation, data were available only for the ADHD group, and separate models were used to predict the modulation in each of the three parietal ROIs (right IPS, left IPS, left TPJ). To predict behavioural response inhibition task performance, separate models were run for the ADHD and control groups. Thus, five regression models were run in total.

To limit the number of predictors in each regression model and lower the risk of over-fitting, we determined potential predictors to include in the models using Pearson’s correlation coefficients: we first calculated partial correlations between all dMRI (FA, AD, RD, MD and volume) metrics and the outcome measures, controlling for sex and age. Then, the variables which were correlated with the outcome at a relaxed level of significance (*p* <.10) were included as potential predictors in the corresponding stepwise regression^60^. In all stepwise regressions, we controlled for age and sex by including them as potential predictors. Collinearity between potential predictors was tested using the variance inflation factor, but no correlation between any predictor variables was severe enough to require attention.

Finally, for tests of difference we corrected for multiple comparisons using Benjamini and Hochberg’s False Discovery Rate, applied at the tract level^61^.

## Results

### Group differences in WM properties

**Fig. 3:**
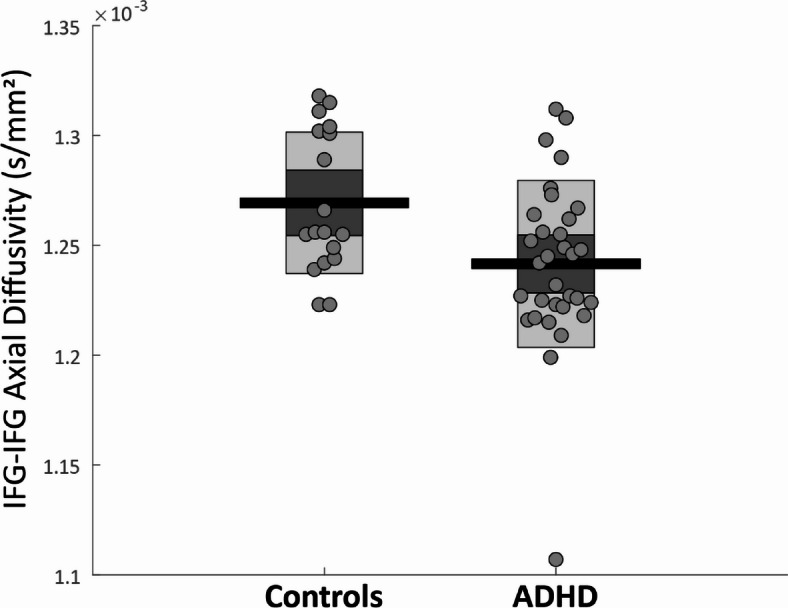
Mean axial diffusivity of the WM tract connecting the right and left IFG was significantly lower in ADHD than in controls. The group difference remained significant when removing the extreme data point from the ADHD group. Horizontal black lines denote group mean, bars denote 95% confidence intervals, and the central dark area of the bar denotes 1 SD around the mean. Circles represent individual participants.

We first tested the differences between groups for the five metrics (volume, FA, MD, AD and RD) in each of the four tracts. AD in the IFG-IFG tract was significantly lower in ADHD (*n* = 32, mean = 1.25 × 10^−3^ s/mm^2^, sd = 3.80 × 10^−5^) than in controls (*n* = 18, mean = 1.27 × 10^−3^, sd = 3.22 × 10^−5^; randomization test corrected for multiple comparisons *p* =.005; Fig. 3). Since one particularly low score seemed to be driving the effect, we ran this comparison again without this participant, and IFG-IFG AD was still significantly lower in ADHD (*n* = 31, mean = 1.25 × 10^−3^ s/mm^2^, sd = 2.90 × 10^−5^; *p* =.007). No other group differences reached significance (*Table S3*).

### Predicting parietal functional modulation by inhibitory demand in ADHD from WM properties

**Fig. 4:**
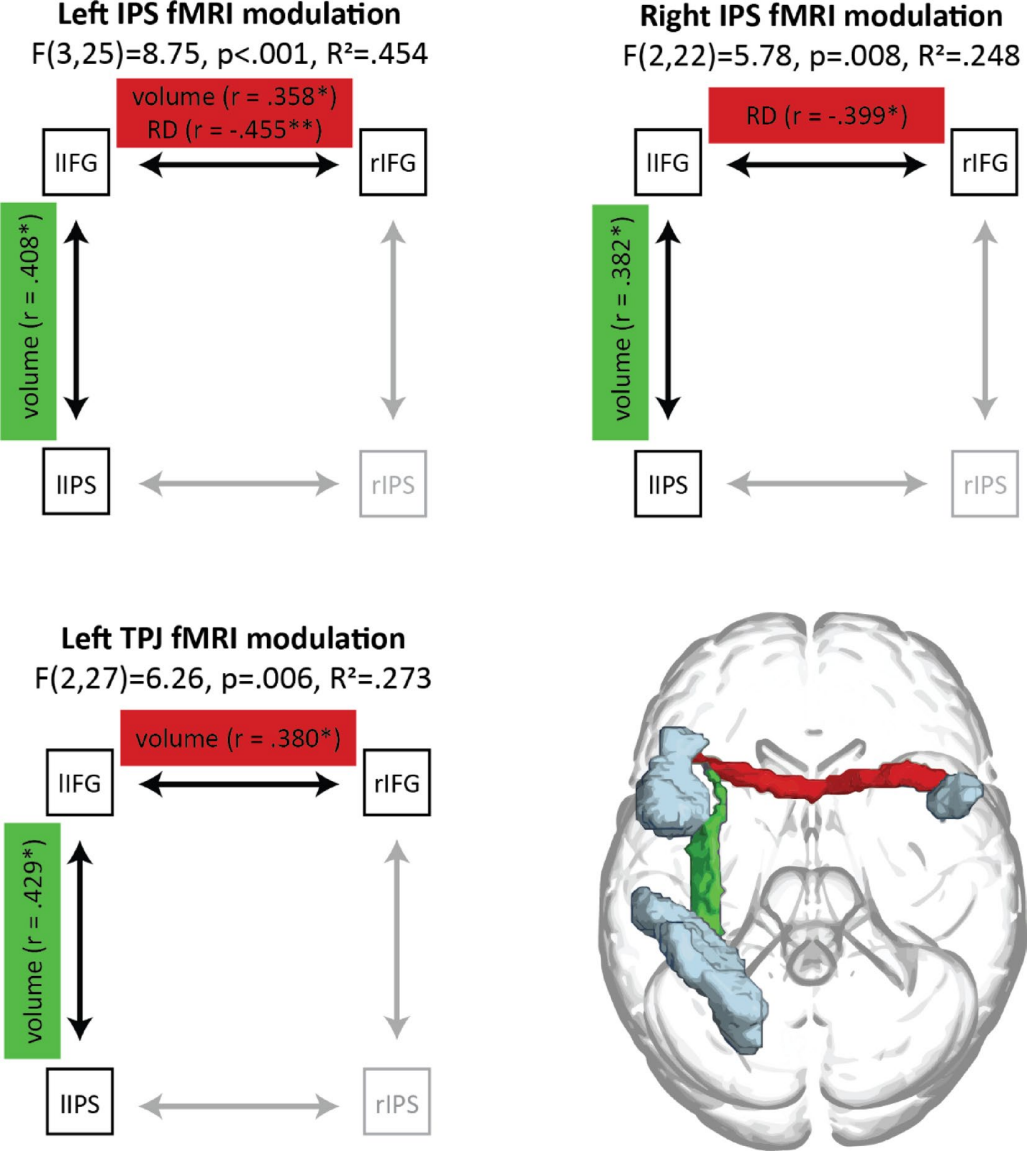
Summary of stepwise regression models predicting fMRI parietal modulations during inhibitory demand in ADHD.

To assess whether structural properties could underlie functional activation during inhibition in ADHD, we used stepwise regression models. Potential structural predictors were determined using the procedure outlined in Sect. 2.4, resulting in four predictors consisting of volume and RD from the IFG-IFG and left IFG-IPS tracts. Sex and age were also included in the models. Three separate stepwise regression models were run with activation in the left IPS, left TPJ and right IPS as the outcome measure. Following multiple comparison correction, all three models were significant, indicating that WM metrics of the IFG-IFG and left IFG-IPS tracts predicted parietal functional modulation by inhibitory demand (Fig. 4; *Table S4*).

Specifically, left IPS functional modulation () was significantly predicted (F(3,25) = 8.750, *p* <.001, R^2^ = 0.454), by the volumes of the IFG-IFG tract (β = 0.358, *p* =.017) and left IFG-IPS tract (β = 0.408, *p* =.007) both contributing positively, and by the IFG-IFG tract’s RD (β = − 0.455, *p* =.003), which contributed negatively (see *Figure *S1 for individual metric visualisation).

Right IPS functional modulation was also significantly predicted (F(2,27) = 5.775, *p* =.008, R^2^ = 0.248). The left IFG-IPS tract volume again positively predicted right IPS modulation (β = 0.382, *p* =.025;), and the IFG-IFG tract RD again negatively predicted right IPS modulation (β = − 0.399, *p* =.020; see *Figure *S1 for individual metric visualisation).

Finally, left TPJ functional modulation was also significantly predicted (F(2,26) = 6.264, *p* =.006, R^2^ = 0.273). Here too, volumes of the IFG-IFG tract (β = 0.380, *p* =.026;) and left IFG-IPS tract (β = 0.429, *p* =.013) positively predicted left TPJ modulation (see *Figure *S1 for individual metric visualisation).

### Predicting response Inhibition performance in ADHD and controls from WM properties

To investigate the relationship of WM properties to response inhibition performance in the ADHD and control groups, we again used stepwise regressions. Potential predictors were determined using the procedure outlined in Sect. 2.4. All of the following results survived multiple comparison correction.

**Fig. 5:**
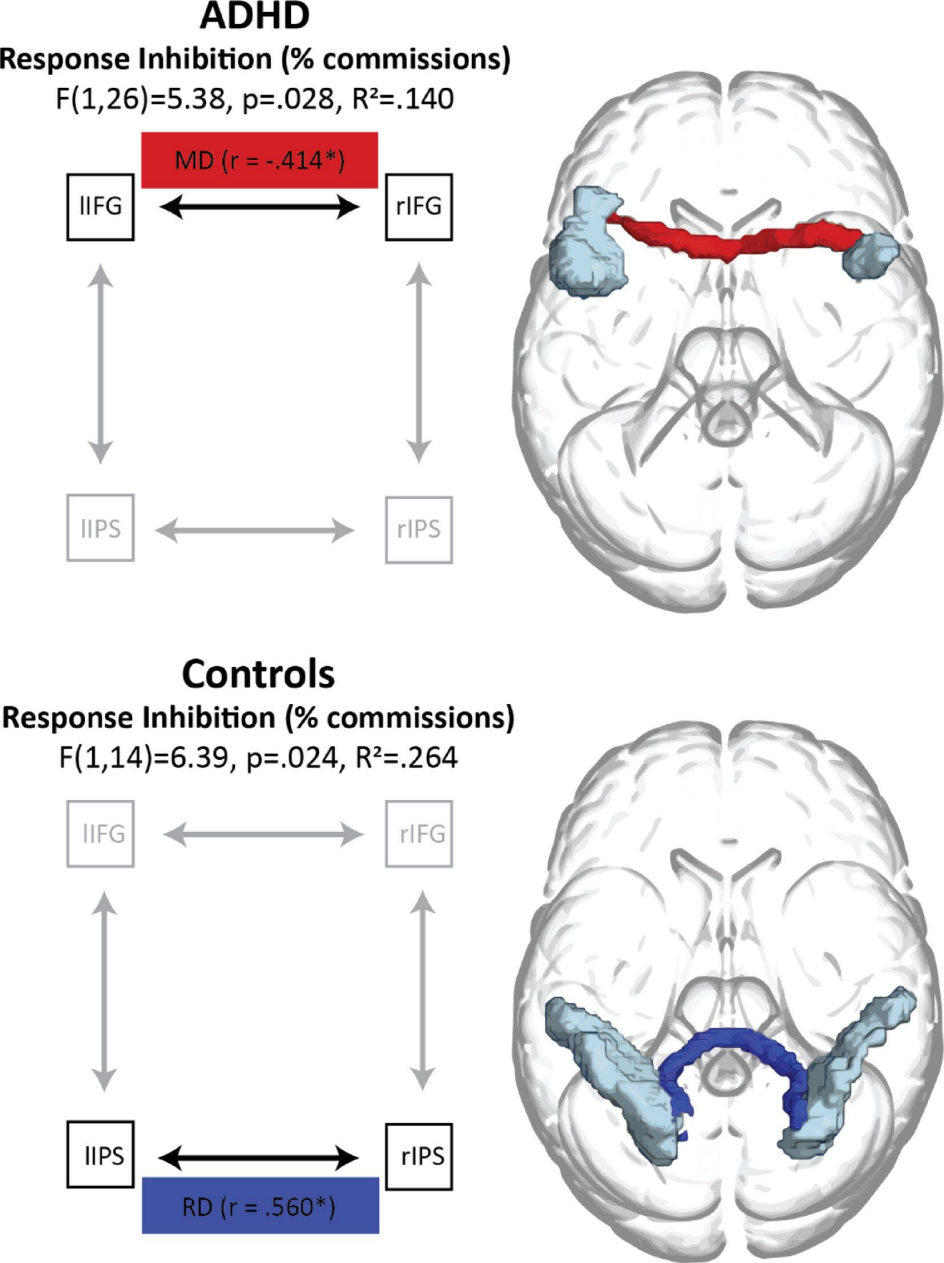
Summary of stepwise regression models predicting response inhibition performance in ADHD and in controls. Data availability statement. The data that support the findings of this study are available from the corresponding author, [DS], upon reasonable request.

Significant models predicted response inhibition performance in each group, but by different structural properties in different tracts (Fig. 5; *Table S5*): In the ADHD group, IFG-IFG tract MD was significantly predictive of response inhibition performance (F(1,26) = 5.380, *p* =.028, R^2^ = 0.140), such that higher IFG-IFG tract MD was associated with better response inhibition performance (lower commission errors): β = − 0.414, *p* =.028 (see *Figure S2a*). This finding was not replicated in controls (see *Figure S2b*).

In controls, response inhibition performance was significantly predicted by IPS-IPS tract RD: F(1,14) = 6.388, *p* =.024, R^2^ = 0.264. The significant contribution made by IPS-IPS tract RD was in a positive direction, with higher IPS-IPS tract RD predicting worse response inhibition performance in controls: β = 0.560, *p* =.024 (see *Figure S2c*). This finding was not replicated in ADHD (see *Figure S2 d*).

Since the significant predictors differed between the models for ADHD and controls, we directly compared the strength of predictor-outcome correlations between groups, using Fisher’s r to z transformation. We found that the relationship between IPS-IPS tract RD and response inhibition performance was indeed stronger in controls (*r* =.544, *p* <.005) than in ADHD (r_s_(28) = − 0.252, *p* =.201; Z = −2.6, *p* <.005), but there was no significant group difference in the correlation between IFG-IFG tract MD and response inhibition performance (controls: r_s_(15) = 0.290, *p* =.294; ADHD: *r* =.125, *p* >.05; Z = 0.52, *p* =.345).

### Examining potential motion confounds

Although we applied several steps of data cleaning in the dMRI analyses (*see* Sect. 2.3.2.2), some residual motion artefacts might still be present and impact the results^62^. As motion is typically higher in ADHD, to ensure structural measures had not been confounded by in-scanner movement we assessed in-scanner absolute and relative motion metrics. There was no significant difference between groups’ motion metrics, and neither metric was correlated with any of the dMRI metrics (*Tables S6-S7*). Thus, it is unlikely that in-scanner motion confounded the current findings.

### Examining the potential role of global WM metrics

To ascertain the specificity of our findings to the IPS-IFG tracts, we repeated the analysis with global WM metrics. Voxelwise analysis was carried out using TBSS (Tract-Based Spatial Statistics; see *Sect. 2.9*). FA and diffusivity metrics were averaged across the skeleton mask, and served as predictors in regression models, with fMRI parietal modulation as the outcome measure, as in the main analyses. However, none of the correlations between global WM metrics and fMRI modulation reached the threshold required to be included as potential predictors, as per Sect. 2.4 (*Table S8*). Thus, there is no evidence to support the notion that non-specific WM properties underly the observed link between the IFG-IFG and left IFG-left IPS tracts and parietal engagement in response inhibition.

It is worth noting that we also investigated the ability of white matter metrics to predict ASRS scores, but this analysis yielded no significant results.

## Discussion

Recent studies from our group pointed to a specific role of functional circuitry between the IFG and the IPS in deficient response inhibition in ADHD^28,29^. In the present study we investigated whether this functional connectivity is also reflected in structural connectivity differences. If the functional differences previously reported are coupled with structural ones it can point to a systematic mechanism that underlies deficient response inhibition in ADHD, and is therefore less likely to result from differences in strategy or engagement of top-down processes. Using diffusion-weighted MRI and seed-based probabilistic tractography, we assessed the WM properties of four tracts connecting the IFG and the IPS bilaterally. This identified the importance of transcallosal structural connectivity (volume and diffusivity) between the left and right IFG as well as of a left frontoparietal tract (overlapping with the SLF) in response inhibition in ADHD. On a group level, there were minimal differences between ADHD and control participants in the WM properties (lower AD) of the IFG-IFG tract. However, on an individual differences’ level within the ADHD group, WM properties of this tract were significantly associated with both the engagement of the parietal regions during task fMRI (volume and RD) and behavioural performance in a response inhibition task (MD).

Some evidence of a role for transcallosal IFG to IFG connectivity in ADHD was provided by lower AD in ADHD compared to controls. Lower AD has been proposed to reflect reduced calibre of, or injury to, axons, or reduced coherence in axon orientation^63^. However, the anatomy which underpins AD and thus the implication of lower AD is debated^64^. Our findings of atypical IFG-IFG AD in ADHD are in line with reports that the IFG is a key node for cognitive and attentional control, whose activity is impaired in ADHD^14,65–67^. Our results also implicate the IFG-IFG tract’s structure specifically in the context of response inhibition. We found that increased IFG-IFG RD was partly linked with lower left and right IPS modulation by inhibitory demand as measured with fMRI. As with AD, the anatomical underpinnings of RD are debated, but increased RD has been proposed to reflect a loss of myelin, loss of axons, or reduced axonal packing density^63^. In addition to the tract’s RD, increased IFG-IFG tract volume was significantly associated with greater left IPS and left TPJ fMRI modulation by inhibitory demand (i.e., modulation that is more similar to that observed in control participants). Factors proposed to drive an alteration in WM volume include number of axons, thickness of myelin around axons, number of axons with a myelin sheath, axonal branching and axonal crossing^68^.

Importantly, IFG-IFG WM properties were not only linked with functional engagement of parietal nodes for increased inhibitory demand, but also behavioural response inhibition performance in a Go/No-go task among participants with ADHD. Increased IFG-IFG tract MD (the average of AD and RD) was associated with better response inhibition performance. Notably, this was not the case for the control group. Instead, their response inhibition performance was linked with the RD of the parietal tract connecting the left and right IPS. These differential results suggest that response inhibition may be executed differently in individuals with and without ADHD. The IFG is associated with cognitive and attentional control^69,70^ and involved in a broad range of other cognitive functions as one of the key domain-general areas in the brain^71^. The fact that individual differences in the IFG-IFG tract in ADHD are associated with behavioural performance may suggest that difficulties in response inhibition stem from an alteration in a more general cognitive function (e.g. context monitoring), or that during response inhibition a broader and less specialised neural network is recruited.

The IFG-IFG tract in the current study crosses between the hemispheres through the anterior part of the corpus callosum. Our findings are consistent with previous reports highlighting lower corpus callosum volume in children with ADHD, and its association with worse sustained attention and attentional control^72,73^. AD in the corpus callosum has also been implicated in ADHD, with increased AD linked with greater symptom severity, and poorer attention and working memory^74^. Thus, the current findings of atypical IFG-IFG connectivity in the more severe ADHD participants may be related to general corpus callosum atypicalities in ADHD and their effect on interhemispheric communication. The fact that the interhemispheric parietal tract was not implicated in ADHD may point to a more specific atypicality in the frontal tract or in the genu of the corpus callosum through which this tract passes. Future research using spatial tract profiling and corpus callosum segmentation is required to further clarify this issue.

The left hemisphere fronto-parietal tract connecting the IFG and the IPS was also related to functional modulation by inhibitory demand in ADHD. Increased left IFG-IPS tract volume was associated with increased recruitment in the left IPS, right IPS, and left TPJ during increased inhibitory demand. This supports the notion that IFG-IPS connectivity is important in ADHD for engaging parietal cortices when response inhibition demand increases^29^. Interestingly, several studies using intracranial EEG and direct electrical stimulation, pointed to an overlap in the timestamp of IPS and IFG involvement in response inhibition, suggesting that IFG/IPS processes run separately but in parallel during inhibitory demand^75–77^. This suggestion runs counter to an alternative hypothesis which proposes that the two regions do not behave separately, but that the inferior frontal cortex inhibits down-stream parietal activity during inhibitory demand^78^. Our findings, where the structural integrity of the pathway between the IFG and IPS is predictive of the recruitment of the IPS in high inhibitory demand, lend support to the latter theory. If the IFG and IPS were separately involved here, we would not expect the structural pathway between them to play a role in the involvement of the parietal node. Thus, the findings we report are more in line with the notion that the inferior frontal cortex modulates down-stream parietal activity during inhibitory demand.

The IFG-IPS tract makes up, in part, the ventrolateral subdivision of the superior longitudinal fasciculus (SLF III). Structure-function relationships between the SLF and specific cognitive functions are yet to be fully elucidated, but associations have been suggested with perceptual organization and working memory^79^, sustained attention^80^, and importantly for the interpretation of current results, response inhibition. Previous research has also implicated WM properties of the SLF in children and in adults with ADHD^81–83^. Our findings suggest the left tract, but not the right tract, is important in response inhibition. This may be surprising given the central role of the right IFG, specifically, in previous studies of response inhibition^84,85^. However, since our findings also demonstrate a role for the IFG-IFG tract, they do not detract from the involvement of the right IFG in the inhibition-related circuitry. Furthermore, our findings are consistent with studies highlighting the role of the left IFG in inhibition^86,87^ and those indicating that its integrity is critical for successful implementation of response inhibition^88^.

In this discussion, we have implicitly considered that structural metrics underlie functional activation and subsequently cause response inhibition performance variation. However, structure-function relationships are known to be bidirectional, and training has been shown to induce changes in WM properties^68^. For example, increased FA in somatosensory and visual cortices followed tactile braille reading training^89^, increased FA in the right IPS followed juggling training^90^, and lower MD in the left arcuate fasciculus followed intensive reading intervention^91^. The findings of the current study suggest that the IFG-IFG and IPS-IFG WM tracts could be used as potential targets to study intervention outcomes. Future research may investigate the plasticity of this neural circuit, perhaps via training. Several training regimes have been demonstrated to improve executive functioning and inhibitory control, including computerized cognitive training^28,92^, mindfulness practice^93^ and neurofeedback^94^, but none of the existing studies tracked induced changes in structural brain properties.

Historically, there has been debate about whether response inhibition is predominantly lateralized to the right hemisphere. Some influential models argue for a right-lateralised network in response inhibition^95^, while others suggest that the process involves a more holistic, transhemispheric network throughout the brain^13,70^. Our results, particularly highlighting the role of the left IFG-left IPS pathway and the right IFG-left IFG pathway, suggest that response inhibition is not exclusively right-lateralized. Instead, it appears to involve a broader, transhemispheric neural network. This finding aligns with the notion that response inhibition is not confined to one hemisphere but rather is supported by a dynamic interplay between both hemispheres.

It is worth noting that, in this study, we chose to focus on response inhibition performance outside the scanner, as we believe it provides a more ecologically valid measure of participants’ inhibitory control abilities. Prior research has shown modest correlations between performance inside and outside the scanner, but also notable differences, such as increased reaction times in the scanner and increased commission errors in the lab^30,96,97^. Given that real-world response inhibition occurs outside the constraints of an MRI environment, we argue that lab-based measures offer a more meaningful link between white matter properties and behaviour.

There are a number of limitations to the current study. First, dMRI data was collected using relatively basic acquisition parameters: a single b-value limits the robustness of cross-fibre modelling, relative to current state-of-the-art multi-shell acquisitions. As no opposite phase encoding direction scan was acquired, motion correction could not include an estimation of motion’s impact on susceptibility-induced distortions. Whilst this step is not mandatory, it is recommended and could be specifically important when one group of participants is more prone for motion, as is the case for ADHD^98–100^. We addressed this by strict preprocessing quality control and excluded data flagged for motion. In the retained data, there was no significant group difference in in-scanner motion, and motion parameters did not correlate with any WM metrics. Thus, although distortion correction could have been beneficial, we believe the current findings are not driven by motion artefacts. Another relevant point to mention here is the use of 24 h washout period for our ADHD participant. While this period is typically enough for many regularly used psychostimulants it might not be enough to eliminate effects of slow-release medications. Since we did not have information about specific medications and dosage for each participant we could not directly test if this played a role in our results. However, since we did not find differences between participants according to their general use of medication, we argue that the risk here is relatively small. Finally, we acknowledge the relatively small sample size of the current study (especially in the control group) that may limit the replicability of our findings.

To conclude, the current study demonstrated a role for WM structure in the atypical recruitment of parietal nodes in ADHD during response inhibition. Microstructural properties of the cross-hemispheric IFG-IFG tract and the left fronto-parietal IFG-IPS tract were linked not only with fMRI activation but also with response inhibition performance in ADHD, as measured behaviourally (outside the scanner). These findings highlight deficient top-down control brought about by maladaptive connectivity as a mechanism of inhibition difficulty in ADHD.

## Electronic supplementary material

Below is the link to the electronic supplementary material.

Supplementary Material 1

## Data Availability

The data that support the findings of this study are available from the corresponding author, [DS], upon reasonable request.

## Declarations

### Permission to reproduce material from other sources statement

Permission to reproduce material from other sources was obtained where applicable.
